# Validating candidate endpoints for intermediate age-related macular degeneration trials in a multi-centre setting—lessons from the MACUSTAR study

**DOI:** 10.1038/s41433-024-03568-2

**Published:** 2025-02-05

**Authors:** Jan Henrik Terheyden, Hannah M. P. Dunbar, Steffen Schmitz-Valckenberg, Charlotte Behning, Cecília Martinho, Ulrich F. O. Luhmann, Marlene Saßmannshausen, Anna Lüning, Alexandra Miliu, Inês Dinis Aires, Pier Giorgio Basile, Joana Batuca, Matthias Schmid, Klaus-Peter Moll, Nadia Zakaria, Adnan Tufail, Alison Binns, David P. Crabb, Sergio Leal, Robert P. Finger, Frank G. Holz, on behalf of the MACUSTAR consortium, H. Agostini, I. D. Aires, L. Altay, R. Atia, F. Bandello, P. G. Basile, J. Batuca, C. Behning, M. Belmouhand, M. Berger, A. Binns, C. J. F. Boon, M. Böttger, J. E. Brazier, C. Carapezzi, J. Carlton, A. Carneiro, A. Charil, R. Coimbra, D. Cosette, M. Cozzi, D. P. Crabb, J. Cunha-Vaz, C. Dahlke, H. Dunbar, R. P. Finger, E. Fletcher, M. Gutfleisch, F. Hartgers, B. Higgins, J. Hildebrandt, E. Höck, R. Hogg, F. G. Holz, C. B. Hoyng, A. Kilani, J. Krätzschmar, L. Kühlewein, M. Larsen, S. Leal, Y. T. E. Lechanteur, D. Lu, U. F. O. Luhmann, A. Lüning, N. Manivannan, I. Marques, C. Martinho, A. Miliu, K. P. Moll, Z. Mulyukov, M. Paques, B. Parodi, M. Parravano, S. Penas, T. Peters, T. Peto, S. Priglinger, R. Ramamirtham, R. Ribeiro, D. Rowen, G. S. Rubin, J. Sahel, C. Sánchez, O. Sander, M. Saßmannshausen, M. Schmid, S. Schmitz-Valckenberg, J. Siedlecki, R. Silva, E. Souied, G. Staurenghi, J. Tavares, D. J. Taylor, J. H. Terheyden, A. Tufail, P. Valmaggia, M. Varano, A. Wolf, N. Zakaria

**Affiliations:** 1 Department of Ophthalmology, University Hospital Bonn, Bonn, Germany; 2 UCL Institute of Ophthalmology, University College London, London, United Kingdom; 3 Moorfields Eye Hospital NHS Foundation Trust, London, UK; 4 GRADE Reading Center, Bonn, Germany; 5 John A. Moran Eye Center, Department of Ophthalmology and Visual Sciences, University of Utah, Salt Lake City, UT USA; 6 Institute for Medical Biometry, Informatics and Epidemiology, Medical Faculty, University of Bonn, Bonn, Germany; 7 Association for Innovation and Biomedical Research on Light and Image, Coimbra, Portugal; 8 Roche Pharmaceutical Research and Early Development, Translational Medicine Ophthalmology, Roche Innovation Center Basel, Basel, Switzerland; 9 European Clinical Research Infrastructure Network, Paris, France; 10 Novartis Institutes for Biomedical Research, Cambridge, MA USA; 11 Division of Optometry and Visual Science, School of Health Sciences, City, University of London, London, UK; 12 Bayer Consumer Care AG, Basel, Switzerland; 13 Department of Ophthalmology, University Hospital Mannheim & Medical Faculty Mannheim, University of Heidelberg, Mannheim, Germany; 14 Department of Ophthalmology, Medical Center, Faculty of Medicine, University of Freiburg, Freiburg im Breisgau, Germany; 15 Department of Ophthalmology, University Hospital of Cologne, Cologne, Germany; 16 Centre hospitalier national d’ophtalmologie des Quinze-Vingts, Sorbonne University, Paris, France; 17 Scientific Institute San Raffaele, University Vita Salute, Milan, Italy; 18 Portuguese Clinical Research Infrastructure Network (PtCRIN), NOVA Medical School Universidade Nova de Lisboa, Lisbon, Portugal; 19 Institute for Medical Biometry, Epidemiology and Informatics, University Hospital Bonn, Bonn, Germany; 20 Department of Ophthalmology, Rigshospitalet, Copenhagen University Hospital, Glostrup, Denmark; 21 Department of Ophthalmology, Leiden University Medical Center, Leiden, The Netherlands; 22 Bayer, Clinical Sciences Experimental Medicine, Wuppertal, Germany; 23 School of Health and Related Research, University of Sheffield, Sheffield, UK; 24 Institut de la Vision, Paris, France; 25 Department of Ophthalmology, Centro Hospitalar Universitário de São João, Porto, Portugal; 26 Novartis Pharma AG, Basel, Switzerland; 27 ZEISS Medical Technology, Carl Zeiss AG, Oberkochen, Germany; 28 Department of Biomedical and Clinical Sciences, Universita degli Studi di Milano, Milan, Italy; 29 Department of Ophthalmology, University Clinic Köln, Köln, Germany; 30 Department of Ophthalmology, Cheltenham General Hospital, Gloucestershire Hospitals NHS Foundation Trust, Cheltenham, UK; 31 Augenzentrum am St. Franziskus-Hospital, Münster, Germany; 32 Department of Ophthalmology, Radboud University Medical Center, Nijmegen, The Netherlands; 33 Optometry and Visual Sciences, School of Health Sciences, City, University of London, London, UK; 34 Centre for Experimental Medicine, Royal Victoria Hospital, Belfast, Northern Ireland UK; 35 Department of Ophthalmology, Donders Institute for Brain, Cognition and Behaviour, Radboud University Medical Center, Nijmegen, The Netherlands; 36 Department of Ophthalmology, University of Ulm, Ulm, Germany; 37 Bayer AG, Leverkusen, Germany; 38 Department of Ophthalmology, University clinic Tübingen, Tübingen, Germany; 39 Ophthalmology Translational Medicine, Novartis Institutes for Biomedical Research, Cambridge, MA USA; 40 Roche Pharmaceutical Research and Early Development, Translational Medicine Ophthalmology, Roche Pharma Research and Early Development, Roche Innovation Center Basel, Basel, Switzerland; 41 Novartis Pharma, University of Southern California, Los Angeles, CA USA; 42 Institut de la Vision, Sorbonne Universités, Paris, France; 43 Ospedale San Raffaele, Milan, Italy; 44 IRCCS-Fondazione Bietti, Rome, Italy; 45 Centre for Public Health, School of Medicine, Dentistry and Biomedical Science, Queen’s University Belfast, Northern Ireland, UK; 46 Department of Ophthalmology, Ludwig-Maximilians-University, Munich, Germany; 47 Sheffield Centre for Health and Related Research, University of Sheffield, Sheffield, England UK; 48 Department of Visual Neuroscience and Function, University College London Institute of Ophthalmology, London, UK; 49 Institut Hospitalo-Universitaire FOReSIGHT, Sorbonne Universite, Inserm, Quinze-Vingts Hopital de la Vision, Paris, France; 50 Diagnostic Image Analysis Group, Radboud University Medical Center, Nijmegen, The Netherlands; 51 Department of Ophthalmology, Hospital Intercommunal de Creteil, University Paris Est Creteil, Creteil, France; 52 University Eye Clinic Luigi Sacco Hospital, Milan, Italy; 53 Department of Ophthalmology, University of Basel, Basel, Switzerland

**Keywords:** Prognostic markers, Retinal diseases

## Abstract

For the conduct of future interventional age-related macular degeneration (AMD) trials, the availability of clinical study endpoints is key. However, no endpoints have been accepted by regulators for evaluation of treatment for intermediate (i) AMD, i.e. the AMD stage at highest risk of developing irreversible geographic atrophy or macular neovascularization. The European MACUSTAR consortium has recruited more than 700 individuals to develop and validate structural, functional and patient-reported endpoints, enabling future iAMD trials based on a prospective observational, multi-centre cohort study. Reliably assessing candidate endpoints in a setting that involves multiple clinical sites across countries comes with a plurality of challenges in the study set-up, quality of data, recruitment of participants and study conduct. Therefore, the MACUSTAR consortium has established a framework that successfully addresses these topics, provides relevant insights into the natural history of iAMD and its sub-phenotypes, and will open new regulatory pathways. The MACUSTAR study is registered on ClinicalTrials.gov under NCT03349801.

## Background

Age-related macular degeneration (AMD) affects more than 196 million people globally and leads to a slow, progressive decline of visual function, ultimately resulting in the loss of macular function [1–3]. Recent therapeutic advances in the late AMD stages highlight the need for effectively treating the condition to reduce the disease burden due to AMD [4, 5]. This is particularly relevant since—despite upcoming treatment options—changes caused by the common dry late stage (geographic atrophy) are irreversible [4–6]. Intermediate AMD (iAMD) is characterized by the presence of large drusen and/or the presence of pigmentary abnormalities [7] and directly precedes these irreversible changes. Therefore, iAMD is considered a relevant target condition in drug development. Clinical validation of pharmaceutical innovations, however, is limited by the availability of validated and accepted clinical trial endpoints [6].

The MACUSTAR consortium consists of 13 partners from academia and industry who have aligned to develop and validate endpoints for future iAMD trials [8]. For this purpose, we have set up and are conducting a multi-centre cohort study on iAMD, neighbouring disease stages and healthy controls, at 20 study sites in 7 European countries (Denmark, France, Germany, Italy, Netherlands, Portugal, and the United Kingdom) [9]. Five study sites are academic core partners within the MACUSTAR consortium, the other sites are affiliated with the consortium and members of the European Vision Clinical Research Network (EVICR.net). These combined efforts have led to the successful recruitment of a study cohort with more than 700 individuals at mostly early AMD stages, enabled the development and validation of standardized test procedures for future multi-centre trials in ophthalmology, and the continued generation of valuable scientific results for the technical evaluation of clinical trial endpoints.

In this article, we have summarized the most important lessons of the MACUSTAR consortium regarding setting up a large-scale multi-centre cohort study, recruitment of multi-language participants, study conduct, and interactions with regulatory bodies.

## Study set-up

The MACUSTAR consortium has set up a comprehensive observational multi-centre cohort study spanning the natural history of early and iAMD over a period of up to six years to develop and validate clinical trial endpoints [8]. The study consists of a cross-sectional part and a longitudinal part [9], which was informed by regulatory recommendations on the development of endpoints [10]. Therefore, MACUSTAR evaluates the reliability, validity, responsiveness to change and clinical significance of morphological, functional and patient reported (PRO) outcome measures [10], all of which criteria are required for the formal qualification of clinical trial endpoints. A particular focus of the MACUSTAR study that sets it apart from similar studies is on the collection of patient-relevant data, which is highly endorsed for eye conditions by international regulatory agencies. The assessments are conducted in individuals across the AMD severity spectrum (early, iAMD, late AMD) and similarly aged control participants with no AMD and across different time points.

The cross-sectional part of MACUSTAR was designed to assess test-retest reliability of candidate endpoints in a multi-centre study setting and has additionally generated data on the construct validity and patient-relevance of assessments and testing protocols [11–14]. The study included male and female patients aged between 55 and 85 years willing to provide informed consent and with no, early, intermediate and late AMD, classified according to the Beckman classification, i.e. the most widely used clinical AMD classification system, which provides a scientific basis for the evaluation of tests’ construct validity [7]. To evaluate repeatability, the structural, functional and PRO assessments were performed twice within a 14 ± 7 day timeframe after enrolment in the study [9, 15, 16].

The longitudinal part of MACUSTAR the prognostic validity of candidate endpoints for the progression from intermediate to late AMD, and the responsiveness of functional tests and PROs to longitudinal changes. It includes participants from the early and iAMD cohorts, with a follow-up of up to 6 years (until February 2026). No longitudinal data of the healthy control (no AMD) cohort are collected within MACUSTAR, which may have provided important information on the natural visual function decline in the context of age-related eye diseases in contrast to the disease-specific changes. In the original study protocol, the follow-up duration of the cohort was 3 years, funded by the European Union Innovative Medicines Initiative 2 (IMI2) programme and the European Federation of Pharmaceutical Industries and Associations (EFPIA), with contributions by Bayer, Novartis, Roche and Zeiss [8]. In 2022, the study was extended at no additional cost from the IMI2/EFPIA budget until August 2023, and, in 2023, an additional study extension was funded by the consortium members Bayer, Novartis and Roche.

### Structural assessments

The clinical classification of AMD is mainly based on colour fundus photographs [7], whereas a multimodal approach is required to perform a state-of-the-art risk assessment based on high-resolution retinal imaging technologies [17]. Given this, the MACUSTAR consortium developed imaging and grading protocols as well as a training qualification assessment for spectral-domain and swept-source optical coherence tomography (OCT), including OCT angiography; fundus autofluorescence, including quantitative fundus autofluorescence; and multi-wavelength confocal scanning laser ophthalmoscopy (cSLO), which are all performed in all MACUSTAR participants. Fluorescein angiography (FA) is performed at the discretion of the investigator in the circumstance of suspicion of conversion to neovascular AMD. In addition to this, adaptive optics imaging is performed at certain sites [9]. The multimodal imaging approach and core integration of a single, highly responsive, central reading centre are one of the key design features of the MACUSTAR study that enable highly precise diagnosis and biomarker categorization. Furthermore, the integration of innovative imaging technologies strengthens the relevance of the study dataset over the longer term.

### Functional assessments

Best-corrected visual acuity (BCVA) is not commonly reduced during early AMD stages [18, 19]. For this reason, a broad battery of visual function assessments has been included in the MACUSTAR study [18, 20–22]. Besides BCVA, this includes low-luminance visual acuity, Moorfields acuity test, contrast sensitivity, microperimetry, dark adaptometry and reading speed, all assessed by certified staff. Each site was required to have at least 2 certified technicians for each procedure prior to beginning recruitment. The MACUSTAR consortium has developed standardized testing protocols and training qualification assessments for all of these assessments. To measure limitations of activities of daily living beyond functional reading, an indoor mobility course was developed and navigation performance assessed at two sites in a sub-cohort and during the cross-sectional part of the study. Given the preference of regulatory agencies of patient-relevant over purely anatomical endpoints, the integration of multiple state-of-the-art functional assessments that cover various visual domains is the second key design feature of the MACUSTAR study. Their core integration not only allows for a better understanding of the natural disease history but also guides the qualification of biomarkers and provides pragmatic information on the potential burden of time and patient fatigue which might be posed by the selection of different functional assessments in controlled drug trials.

### Patient-reported outcomes

Vision-related quality of life is reduced by AMD but commonly used PRO instruments have ceiling effects in early and iAMD, lack face validity and show poor psychometric performance [23]. The Vision Impairment in Low Luminance (VILL) questionnaire, implemented in the MACUSTAR study, was developed to assess the low-luminance / low-contrast vision deficit in early AMD stages [24]. A standardized translation and cultural adaptation was a prerequisite of this, given the multi-language setting across the 20 MACUSTAR study sites. This process followed international recommendations and demonstrated inter-cultural equivalence of the questionnaire [14]. The MACUSTAR consortium has developed a PRO administration guideline and implemented the VILL together with the EQ-5D-5L instrument [25] in both parts of the study. In line with the broad collection of visual function assessments, the integration of PROs in MACUSTAR was a key decision to collect patient-relevant data in a study that was designed to inform multi-centre randomized controlled drug trials in the future.

## Recruitment of participants

Participants were recruited to take part in the MACUSTAR study between March 2018 and January 2020, spanning a period of 92 weeks. The recruitment period, initially planned for 48 weeks, was extended mostly due to the availability of devices, ethical approvals, contracting and the necessity of implementing the upcoming European Union General Data Protection Regulation (GDPR). The first participants were screened at different time points at the participating study sites. Continuous recruitment monitoring and communication were key factors to ensure recruitment goals were reached [26]. Recruitment strategies and measures were planned centrally and then implemented across all sites. Based on site-specific recruitment monitoring sheets provided on a weekly basis, it was possible to identify localised difficulties early on. Nonetheless, recruitment was not linear over time and one of the key learnings was the importance of incentivizing continuously during the recruitment period for a multi-centre study. Communication activities targeted different study personnel, including project managers, data managers, principal investigators, technicians, study coordinators and clinical monitors, and involved virtual (teleconferences, study newsletter, individual phone calls) and in-person meetings (investigator meeting at annual EURETINA conference, scientific advisory board meeting at Association for Research in Vision and Ophthalmology [ARVO] conference).

Recruitment of the main target group, iAMD participants, was successful in 76% of screenings (based on participants from pre-screening lists who were finally determined to be eligible; exclusion was based on e.g. AMD diagnosis and stage, comorbidities), with an average rate of 0.6 ± 0.9 screenings per week [26]. Factors that influenced weekly screening rates included facilitator teleconferences with site investigators and the barriers of approaching a pre-aligned recruitment target and impact of public holidays [26].

Besides the availability of study participants, the selection and initiation of participating sites, and the availability of staff and devices were important pre-requisites for the start of recruitment activities. The MACUSTAR consortium was already formed during the application to the Innovative Medicines Initiative (IMI) 2 funding, and built upon existing networks in the ophthalmology community (including professional associations, and previous collaborations in other research programmes). Given that the recruiting sites are tertiary referral centres, the majority of study equipment was available before the start of the study and devices not available beforehand were bought and shipped to the sites before the local site initiation visit. The availability of equipment was closely monitored in weekly to biweekly calls during the initiation phase to be able to start recruiting at all sites as early as possible. Since iAMD patients are not typically followed up at referral centres, all sites were asked to prepare pre-screening lists and provide respective numbers to the study sponsor early on. Nonetheless, the recruitment activities after the site initiation visits differed noticeably between the sites, due to the scheduling of appointments (resource allocation for other, already running studies), staff requirements (study certification), and the ad-hoc availability of participants from the existing pre-screening lists. This was solved by individual calls to find tailored solutions and the preparation of a consortium recommendation for the scheduling of appointments where a reduced number of different staff members involved with the examination of an individual patient was recommended (i.e., one or few study nurses run all tests in a participant).

## Study conduct

**Fig. 1:**
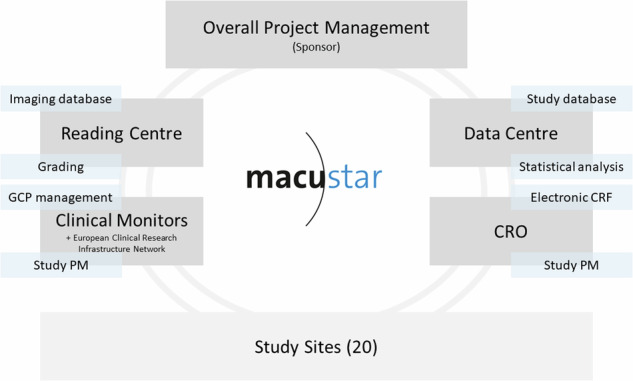
Organizational flow chart. Early established data pathways and quality control mechanisms are enablers of the results generated by the MACUSTAR study. CRF case report form, CRO, clinical research organization (Association for Innovation and Biomedical Research on Light and Image), GCP good clinical practice, PM project management.

From the experience of the MACUSTAR consortium, rigorous, ongoing data management and quality assessment are important drivers of quality during a high-impact, clinical study involving multiple sites and stakeholders (Fig. 1). All study procedures are specifically scheduled and performed in equipped study centres independently from clinical care. Throughout the study, a risk assessment plan with corresponding contingency and mitigation measures has been followed and continuously updated. The first step of ensuring collected data are reliable was the provision a set of standard operating procedures for structural, functional and PRO assessments. Throughout the study, this has been accompanied by the online training (re-training, if necessary) of all new technicians, and a certification procedure for imaging and functional data (with re-certification, if necessary), which involves collection of sample data by the candidate technician, subsequently submitted to the central reading centre for quality assessment. A key aspect during the certification procedure is the timely evaluation of the certification results, guaranteed by staff of the central reading centre. A second important aspect to its success has been the provision of individualized feedback based on the testing results in low-performing technicians. Between March 2018 and March 2024, a total of 128 and 93 technicians were certified for the imaging procedures and visual function assessments performed in MACUSTAR, respectively. Re-certification was necessary in a noticeable proportion of cases, e.g. in 37 instances for the Cirrus OCT device (34.6%), 49 instances for the MAIA fundus-controlled perimetry device (53.3%), and 41 instances for the AdaptDx adaptometry device (51.3%). From the consortium’s experience, rolling out a certification procedure requires high staff availability and quick turnaround times to ensure study data collection in the necessary timeframe.

The second contributor to high-quality data in the MACUSTAR study was related to study management. A multi-centre study can include more than one party involved with project management activities. In the MACUSTAR consortium, these are:

- Overall project management, located at the sponsor institution (Department of Ophthalmology, University Hospital Bonn, Germany)
- Clinical research organization project management (Association for Innovation and Biomedical Research on Light and Image [AIBILI], Coimbra, Portugal)
- Clinical monitoring project management (European Clinical Research Infrastructure Network [ECRIN], Paris, France)
- Central reading centre project management (GRADE Reading Center, Bonn, Germany)
- Clinical study coordination team, including equally represented members from academic and EFPIA partners of the MACUSTAR consortium

These parties together are responsible for the oversight of the study, although with different delegated tasks. The original participant-related data was collected and documented at individual clinical sites in accordance with the International Conference on Harmonisation-Good Clinical Practice (ICH-GCP) guidelines, GDPR and the respective national data protection law, and only after giving written informed consent, as approved by the local Ethics Committees. Clinical sites enter data in a pseudonymized form using the MACUSTAR electronic case report form (eCRF), which was designed based on the study protocol of the MACUSTAR study. The data collected in the eCRF include demographics, relevant medical history (e.g. past and/or ongoing ophthalmological and other relevant disease), clinical data on visual function, patient-reported data on visual functioning, imaging data of the ocular fundus (retinal imaging) and genetic data on known AMD risk genes.

The MACUSTAR study data flow includes collection at the study sites, followed by entry of categorical and numeric data onto the electronic case report form (eCRF). This includes information on inclusion/exclusion criteria, visual function and PROs. All imaging data are directly transferred from the clinical site to the central reading centre, using a secure online network provided by GRADE reading centre. Additionally, source files from device-assessed visual function tests (fundus-controlled perimetry, Macular Integrity Assessment [MAIA], iCare, Finland; dark adaptometry, AdaptDx, Lumithera, Poulsbo, WA) are also transferred from study sites to the central reading centre. On a regular basis, per clinical site, according to the data entry process and/or the volume of pending issues, completion rate assessments are performed based on the eCRF and the reading centre databases and reminders sent when indicated. The result of this process has been high overall completion rates over a 3-year period in a multi-centre setting for even visual function assessments that are known to be relatively burdensome and tiring (e.g. microperimetry: 76.6-78.4% completed tests that also passed quality checks; dark adaptometry: 61.7% completed tests that also passed quality checks), even if first performers of the respective test were included. A Data Management Plan was issued to describe the overall concept of data management, data flow and responsibilities in the MACUSTAR clinical study. The results of these assessments are shared with members of the MACUSTAR consortium during regular remote teleconferences, as well as with principal investigators and the MACUSTAR scientific advisory board. The meetings and communication strategies employed at the recruitment phase have been kept throughout the study to ensure the clinical team is kept informed and motivated and that any issues are solved in a timely manner.

Six-monthly monitoring visits are performed by study-specific and country-specific monitors. Besides completion rates, monitors assess protocol compliance at individual sites and the accuracy of eCRF data based on source document sampling. During the COVID-19 pandemic, monitoring visits were performed online in accordance with the restrictions imposed at that time. The clinical research organization’s project management team supervises the monitoring activities and reviews the monitoring reports ensuring that similar criteria are applied across the different counties, issues are solved in a timely manner and an efficient route of communication with the study sponsor is guaranteed.

Due to the complexity of visual function testing being implemented across multiple centres by multiple technicians, and the possibility of data entry errors arising during manual data entry onto the eCRF [27], the MACUSTAR consortium has implemented six-monthly retrospective quality checks of all visual function data available in the preceding 6 months. This not only enables the exclusion of data which are of insufficient quality, according to a series of pre-specified criteria but also enables site-specific problems with data collection to be flagged. Issues identified have included re-training needs for study technicians after >12 months since the initial certification for study procedures (rectified by the MACUSTAR “visual function outcomes” work package providing individual video calls where necessary), and difficulties with the comprehensive dark adaptometry protocol (solved by circulating a “common questions” document as an appendix to the standard operating procedure).

The retinal structural and PRO data also undergo complex mechanisms of quality control. The imaging data collected in the MACUSTAR study are graded, following grading protocols by ≥2 retinal imaging expert graders, including a junior and a senior grader for each eye. Prior to the grading itself, the evaluation process includes a data manager / reader review of image quality. When image quality is considered insufficient, a replication of the respective imaging procedure is requested from the study site. During data analysis, the quality of collected PRO data, including responses to individual items, is evaluated on a statistical basis, as reported previously [14].

Analysis of study data took place after cross-sectional data collection was completed and again after the first phase of the longitudinal part was completed (IMI2/EFPIA-funded phase, median follow-up: 3 years [interquartile range: 2.5 – 3.5]), according to ICH-GCP and to the Good Clinical Data Management Practices. Data from the respective visits available in the eCRF and imaging databases were monitored, cleaned, quality controlled and transferred to the central study database located at the Institute for Medical Biometry, Informatics and Epidemiology (IMBIE), University of Bonn. Here, additional quality check procedures in accordance with the statistical analysis plan of the cross-sectional and longitudinal parts of MACUSTAR were conducted. All the data quality control steps were detailed in the data management plan. The same steps will also be followed after the 6-year review period has been completed by all participants of the longitudinal part of MACUSTAR.

### Published results and implications

While the data collection and analysis are still ongoing, the MACUSTAR study has already produced results that have provided significant insight into the natural history of AMD and will allow future iAMD trials to be planned more efficiently. Both the cross-sectional and longitudinal parts of the MACUSTAR study provide a scientific rationale for further distributing the consortium’s approach of featuring functional endpoints besides anatomical endpoints in multi-centre trials on iAMD. Chart-based and device-based visual function tests including BCVA, low-luminance visual acuity, contrast sensitivity, microperimetry and dark adaptometry were highly repeatable in a multi-centre setting when the newly developed standard operating procedures were used, yielding intra-class correlation coefficients of ≥0.7 in individuals with iAMD. This was generally consistent across different disease stages, including early AMD, iAMD, and late AMD [12, 13]. Nevertheless, the power to discriminate iAMD from no AMD was limited (area under the receiver operating curve: 0.59-0.77), which highlighted the need to better stratify individuals with early AMD stages (early and iAMD) [12, 13]. In line with this, the MACUSTAR study showed a remarkable heterogeneity in visual function across the spectrum of iAMD, which partly overlapped with the no AMD and late AMD disease groups across tests [12, 13]. The largest proportion of individuals with iAMD (71%) yielded a performance in at least one visual function parameter that was worse than 95% of test takers with normal retinal health [28], indicating that visual function deficit in early AMD stages is very common.

The relevance of functional iAMD endpoints beyond well-established structural biomarkers was highlighted by the primary endpoint analysis, where an anatomical biomarker (presence of reticular pseudodrusen) and a functional assessment (mesopic microperimetry pattern standard deviation) were significantly prognostic of the progression from iAMD to late AMD when controlling for age [29]. Further than that, the results from the longitudinal part of MACUSTAR suggest that the presence of a visual function deficit has statistically significant prognostic relevance and is an early indicator of structural progression to late AMD [30].

The MACUSTAR consortium has confirmed several structural risk factors of AMD progression and used innovative imaging modalities to newly develop and further define prognostic biomarkers and trial endpoints. An important foundation of this was the inter-session repeatability of assessments. The cross-sectional study results demonstrated that the reading centre intersession agreement parameters were highest for parameters with clear cut-off values, such as drusen size or presence of large pigment epithelium detachments and underlined the importance of rigorously defining structural parameters. Less precisely defined biomarkers like vitelliform lesions and refractile deposits showed lower intersession agreement, possibly due to less defined criteria and subtle nature of these features [16]. The baseline results furthermore confirmed a significant spatial association between large drusen, Hyperreflective foci, and early OCT stages of atrophy (iRORA/cRORA), particularly in the perifoveal area, supporting the further use of early atrophy stages as study endpoints, potentially [16]. The anatomical baseline results from the MACUSTAR study also laid the foundation for mapping structural features across imaging devices, given that the repeatability across devices is limited [31]. Further development work on algorithms and innovative deep learning approaches based on the MACUSTAR cohort may improve the precision of grading tasks and relieve human graders at reading centres in the future, e.g. in the context of the consensus-defined endpoints developed by the Classification of Atrophy Meeting (CAM) group or of novel biomarkers such as ellipsoid zone reflectivity [32, 33].

Besides functional and anatomical endpoints, the MACUSTAR study further supported that including PRO data needs to be an essential part of future iAMD studies. The VILL questionnaire has been evaluated in terms of various psychometric criteria including repeatability, construct validity, prognostic validity and responsiveness to change over time. Similarly to the functional assessments, intra-class correlations were ≥0.7, supporting the questionnaire’s repeatability [14]. On top of this, the VILL has shown to be significantly prognostic of progression from iAMD to late AMD, when controlling for the same variables included in the primary endpoint model [34]. This highlights further potential uses of the VILL questionnaire in prognostic considerations, which extend beyond its use in the evaluation of patient-relevance of drug efficacy. Besides the PRO instrument, the MACUSTAR consortium has developed and validated a VILL-utility instrument that can be used for health economic evaluations [35].

Lastly, the MACUSTAR study is continuing to generate insights into the association between polygenic AMD risk and structural features, as first published analyses have highlighted [36, 37]. The MACUSTAR consortium also undertakes continues continued collaborations with international scientific consortia, including the reticular pseudodrusen gene consortium [38]. The MACUSTAR biobank will enable future analyses to focus on various additional omics approaches that allow the continuation of the MACUSTAR consortium to perform deep phenotyping and sub-classify iAMD.

### Impact of multi-centre assessment

The multi-centre setting of the MACUSTAR study makes it highly comparable to the settings under which clinical trials are conducted. Regulators consider visual function endpoints particularly patient-relevant [10], and the MACUSTAR study performs a broad spectrum of visual function assessments.

**Fig. 2:**
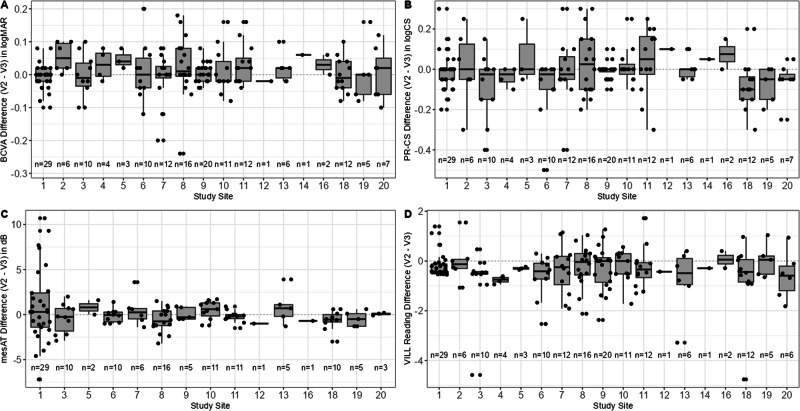
Inter-visit differences in visual function and patient-reported outcomes. Differences between baseline and validation visits across MACUSTAR study sites of exemplary visual function (**A**–**C**) and patient-reported outcome assessments (**D**) across MACUSTAR study sites in individuals with intermediate age-related macular degeneration that participated in the cross-sectional part. Two out of 20 sites did not contribute to the recruitment for the cross-sectional part and are not listed here; specific examination data (e.g. microperimetry, dark adaptometry) of individual sites had to be additionally excluded due to the availability and quality of data. BCVA best-corrected visual acuity, mesAT mesopic average threshold on microperimetry, PR-CS Pelli-Robson contrast sensitivity, VILL Vision Impairment in Low Luminance questionnaire.

The number of participants included at each MACUSTAR study site varied, ranging from 18 to 76 individuals with iAMD (median: 25 participants [interquartile range: 20–32]). Despite high training needs for visual function tests and that numerous sites had not performed the study assessments before the start of MACUSTAR, test repeatability was comparably high among sites where ≥10 examinations were conducted [12]. This holds true for chart-based and device-based visual function tests as well as the VILL questionnaire (Fig. 2) and supports the use of publicly available MACUSTAR protocols in future multi-centre trials [9, 12, 13]. To overcome challenges in the collection of visual function data in studies with multiple clinical sites, there may nonetheless be advantages of excluding first-performing study participants of complex functional tests from such studies or running training sessions with patients. Overall, the standard operating procedures developed in the context of MACUSTAR enable future trials in the iAMD space to be conducted with the knowledge of the reproducibility of the individual assessments, which may make future assessment of test-retest reliability optional.

**Fig. 3:**
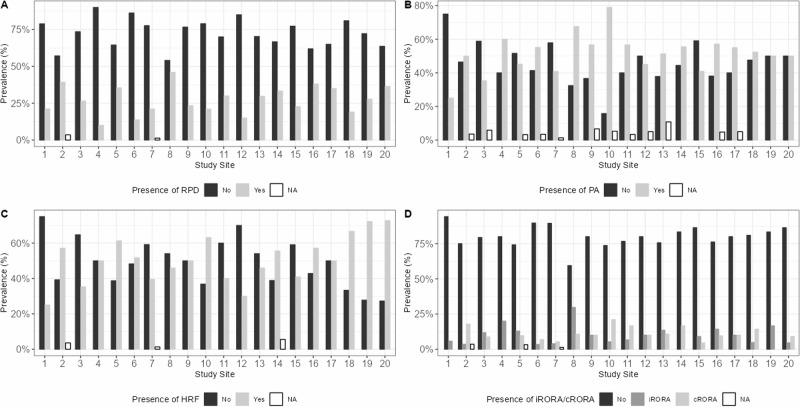
Baseline prevalence of selected structural biomarkers. The prevalence of reticular pseudodrusen (**A**), pigmentary abnormalities (**B**), hyperreflective foci (**C**) and incomplete or complete retinal pigment epithelium and outer retinal atrophy (**D**) noticeably varied ross MACUSTAR study sites in all participants with intermediate age-related macular degeneration.

AMD phenotypes are known to be highly heterogeneous [39]. This is also reflected by the findings of the MACUSTAR consortium [11] and the prevalence of common structural biomarkers noticeably varies across sites (Fig. 3). Considering these biomarkers are prognostic of progressing to late-stage AMD and visual loss, a multi-national, multi-centre setting of future trials investigating pharmaceutical products in the context of iAMD seems compulsory to ensure the external validity of the findings. Even though little is known about the geographic differences in the prevalence of state-of-the-art structural AMD risk biomarkers, recruitment patterns and clinical care pathways might impact the composition of study cohorts that are recruited in a single-centre setting.

## Interaction with regulatory bodies

The MACUSTAR study was specifically designed for the “development of novel clinical endpoints for clinical trials in patients, with a regulatory and patient access intention” [8]. Therefore, the MACUSTAR consortium sought contact with regulatory bodies early in the process. To date, two scientific advice procedures with European (European Medicine Agency, EMA and National Institute for Health and Care Excellence, NICE) and American regulatory bodies (Food and Drug Administration, FDA) have taken place and a third procedure has taken place recently (2024). The consortium has addressed specific issues with regard to study design, the process of developing iAMD endpoints and a treatment indication, as well as the reliability and validity of structural, functional and PRO assessments in future trials. Efforts were generally supported by regulatory agencies and the European Medicines Agency (EMA) provided two letters of support which are both publicly available [40, 41].

In summary, a first discussion meeting (2016) was a joint meeting between the EMA, the United States FDA, the United Kingdom’s NICE and the MACUSTAR consortium. It issued the purpose and design of the MACUSTAR study and supported the general approach taken by the MACUSTAR consortium. The EMA noted the design limitation of natural history studies such as MACUSTAR to investigate the predictive value of a biomarker, given the lack of an interventional arm, whereas they confirmed the study design as appropriate to identify prognostic biomarkers of regulatory relevance [40]. The second discussion meeting (2021) included the EMA and the MACUSTAR consortium and was based on the results of the cross-sectional part of the MACUSTAR study, addressing the repeatability of structural, functional and PRO assessments and the visual function deficit in iAMD as a potential treatment indication. Main outcomes of this meeting from the European regulatory perspective were:

1. Defining a novel treatment indication for functional impairment in iAMD was deemed in principle acceptable,
2. The reproducibility of all presented outcome assessments (structural, functional and PRO measures) using the developed standard operating procedures was supported and
3. The ongoing validation of the VILL questionnaire for use as a PRO in future iAMD trials was encouraged [41].

The results of the third meeting (2024) have not yet been published (November 2024) but further supported the approach taken by the MACUSTAR consortium.

## Research context

Other studies besides MACUSTAR have recruited individuals with iAMD in a multi-centre setting. While they mostly focus on structural outcome assessments with the goal of evaluating therapeutic options and developing structural iAMD biomarkers, MACUSTAR holds the unique position of implementing a broad battery of morphological, functional as well as PRO assessments, allowing for an in-depth phenotype assessment of individuals with iAMD.

### Age-related eye disease studies

The Age-Related Eye Disease Study (AREDS) on the effect of vitamins and antioxidants on progression of AMD and cataracts recruited 3640 individuals for its AMD trial. It involved 11 study centres, assessing BCVA and fundus photographs as functional and structural measures of AMD respectively [42]. The subsequent study, AREDS2 enroled 4203 individuals at a total of 82 study sites and additionally allowed facultative submission of fluorescein angiograms and OCT images [43]. Both AREDS and AREDS2 involved centralized grading of imaging data by a reading centre and a grading protocol, similar to MACUSTAR [42–44]. However, since the availability of multimodal retinal imaging has advanced significantly since AREDS and AREDS2, the development of standard sets of state-of-the-art grading methods was necessary and has been implemented in MACUSTAR [11]. Moreover, no standard operating procedures for visual function assessments and PRO data relevant in the context of iAMD were available from AREDS or AREDS2 and have been developed and published by the MACUSTAR consortium [9, 12, 13].

### Interventional trials

The Laser Intervention in Early Stages of Age-Related Macular Degeneration (LEAD) study investigated the effect of subthreshold nanosecond laser on the progression of iAMD and included 292 individuals at six study centres in Australia and Northern Ireland [45]. It included a multimodal assessment of participants based on structural, functional and PRO assessments (BCVA, low-luminance visual acuity, microperimetry, multimodal imaging, Night Vision Questionnaire, Impact of Vision Impairment Scale). Given the aim of the trial, the investigators did not specifically seek regulatory qualification of endpoints for future trials in the context of pharmaceutical products [10, 45]. The LEAD investigators have specifically analysed the interaction between the treatment effect and study site, given the nature of the intervention but did not identify a significant association (*p* = 0.777) [45].

An interventional phase 2a trial has recently assessed the safety and explored the efficacy of applying risuteganib (Allegro Ophthalmics, San Juan Capistrano, CA), a peptide with an effect on integrin molecules in human retinal pigment epithelial cells, in 45 individuals with iAMD and a BCVA between 20/40 and 20/200 [46]. The study included 39 participants at seven US-sites but did not report any results related to its multi-centre design.

### Observational studies

Two more recent studies also target the lack of endpoints in iAMD. The HONU study is a prospective observational multi-centre cohort study currently enroling 400 iAMD participants with atrophic changes in the non-study eye [47]. Similarly to MACUSTAR, it assesses structure, function and patient-reports in the study cohort but focuses more on individuals at a particularly high risk of geographic atrophy development. This is also reflected by HONU’s primary study objective, i.e. to investigate progression and progression rates to atrophic changes, which provides a highly valuable addition to the more heterogeneous MACUSTAR cohort.

Lastly, the PINNACLE study is a multi-centre observational cohort study investigating individuals with iAMD in one or both eyes. It comprises a retrospective and a prospective part; the latter includes participants recruited at twelve centres in Austria and the United Kingdom [48]. PINNACLE targets the development and validation of biomarkers, specifically using machine learning algorithms, and includes BCVA, low-luminance visual acuity and microperimetry-related functional outcomes [48]. The novel developments of machine learning algorithms in this context nicely complement the broad assessment of structure, function and patient-reports in MACUSTAR.

## Conclusions

The MACUSTAR consortium has designed a study with the aim of developing and validating endpoints for iAMD trials and has developed a comprehensive methodology for assessing structural, functional and PRO measures in the context of multi-centre studies. Published results support the reliability of the approach and suggest that assessment across all three outcome categories is feasible in future multi-centre iAMD trials. Beyond this, protocols developed by the MACUSTAR consortium can serve as guidance for other ophthalmic conditions where novel endpoints are needed. The follow-up of the MACUSTAR study cohort is ongoing and additional evaluation by regulators will be sought in the future to further advance trial endpoint qualification.
